# “It’s a Slippery Slope”: Antecedents of Female-Perpetrated Sexual Abuse of Adolescents in Australian Educational Settings

**DOI:** 10.1177/10790632241290507

**Published:** 2024-10-10

**Authors:** Amanda L. Robertson, Danielle A. Harris

**Affiliations:** 1 Griffith Criminology Institute, 5723 Griffith University, Mt Gravatt, Australia

**Keywords:** institutional child sexual abuse, female perpetrators, educational settings, professional boundaries, gender stereotypes

## Abstract

Sexual victimization of students endures—especially in secondary schools—and women’s perpetration in this setting is increasingly recognized. Nonetheless, our understanding of this population and contemporary cases remains limited, and research in the Australian context is lacking. This study contributes to the evidence base and represents the first Australian study of its kind. We draw on (1) legal documents (*N* = 19) describing 18 established cases of female-perpetrated sexual abuse against 20 adolescent students (aged 13–17 inclusive) and (2) semi-structured expert interviews with professionals possessing specialized experience of female-perpetrated cases involving adolescent students (*N* = 8). These data are integrated in a reflexive thematic analysis to identify the personal, contextual, and situational antecedents of female-perpetrated cases thereby exploring the ‘why’ and ‘how’ of their perpetration. Taken together, the results indicate a key interaction between individual emotional and relational needs, environmental opportunities, and contextual tolerance. Practical implications for targeting and disrupting these dynamics are discussed.

## Introduction

Despite the worldwide attention institutional child sexual abuse (CSA) has attracted in recent years, students continue to be sexually abused by schooling employees. According to a recent American prevalence study, 11.7% of students will experience at least one form of employee-perpetrated sexual misconduct throughout their entire schooling, with 1.4% subjected to serious contact offenses like kissing, touching, and intercourse (Jeglic et al., 2023). Secondary school students—typically aged 13–17 years—are consistently reported to be most at risk of this kind of victimization (Canadian Centre for Child Protection Inc, [CCCP], 2019; Christensen & Darling, 2020; Jaffe et al., 2013).

Though most perpetrators of CSA in schools are male employees, females are also implicated. For instance, women composed 13% of a Canadian sample of abusive personnel (CCCP, 2019) and about 25% of American criminal (Ratliff & Watson, 2014) and noncriminal (Robert & Thompson, 2019) samples of abusive educators. These figures increase when directly surveying American students, who identified female perpetrators in 43% of sexual misconduct cases (Shakeshaft, 2004). Women seem particularly likely to victimize male secondary school students (e.g., Christensen & Darling, 2020). The proportion of cases perpetrated by women varies depending upon sampling methods and inclusion criteria, but their sexual perpetration is clearly not insignificant in schooling contexts.

There is, however, a paucity of empirical enquiry about female-perpetrated CSA in schools, especially in Australia (Proeve et al., 2016). Its general neglect can be partly attributed to pervasive gender biases and socialization processes regarding both offenders and victims. For instance, women are conventionally conceived of as passive recipients of sex rather than initiators, and adolescent boys are regarded as highly sexually motivated (Denov, 2003; Saradjian, 2010). Rather than being viewed as aggressors, there is a puritan nurturer connotation associated with women, one that is predominantly loving and maternal. Female-perpetrated abuse—particularly against boys—is thus often considered less harmful than that perpetrated by men (Denov, 2003; Russell & Gruys, 2022). Consequently, it is rarely viewed as abusive, let alone criminal. This gendered discourse has extended to minimizing and romanticizing media portrayals of female-perpetrated CSA (e.g., Hayes & Baker, 2014). Such stereotypes inhibit boys’ disclosure and likely influence their lower reporting rates (Saradjian, 1996). Moreover, these stereotypes further diminish the real lifelong harm caused by female-perpetrated CSA that male victims report (Christensen, 2021; Denov, 2004; Gerke et al., 2023). Indeed, these studies show that impacts of female-perpetrated CSA have been reported as comparable to—if not *more* damaging than—that perpetrated by men.

There is a clear need to build upon the emergent international studies specifically exploring female educators’ perpetration (e.g., Christensen & Darling, 2020; Steely & Ten Bensel, 2020). Accordingly, our study aims to contribute to the evidence base about women’s sexual abuse of adolescents in schooling environments. To our knowledge, it is the first to examine this phenomenon in an Australian context. We employ a multi-methods qualitative approach to thematically analyze the antecedents to women’s abuse thereby examining the underlying processes and mechanisms to explore the ‘why’ and ‘how’ of their perpetration. Such findings are critical to understanding the enabling personal, situational, and contextual factors that could be targeted in prevention efforts.

## Background

Female and male sexual offenders exhibit difficulties across the same broad domains—cognitive, emotional, relational, and sexual—but they typically manifest in gender-specific ways (Cortoni & Gannon, 2017). Though reported by fewer women than men, women do acknowledge a sexual interest in children (Erkan et al., 2023; Savoie et al., 2021). In the absence of sexual interest however, sex with minors can fulfill women’s other needs like emotional intimacy (Cortoni & Gannon, 2017; DeCou et al., 2015). Indeed, this reflects the underlying premise of the Good Lives Model (GLM) of rehabilitation for male sexual perpetrators: that offending results from seeking or prioritizing to meet various basic human needs in maladaptive ways (e.g., Mallion et al., 2020; Ward & Gannon, 2006).

Early literature on *institutional* perpetrators focused exclusively on men (Smallbone et al., 2008). Initially labelled ‘professional perpetrators’, this group were described as those who use their professional employment to target and abuse children (Sullivan & Beech, 2002). Since then, studies examining perpetration in different institutional settings (not just schools, e.g., religious contexts) have demonstrated variation among the population. Some corroborate predatory perpetrators intentionally seeking out child-related employment to offend (e.g., Leclerc & Cale, 2015; Sullivan & Beech, 2004; Sullivan et al., 2011) but others do not (e.g., Erooga et al., 2012). Rather than entrenched sexual attraction to children, Erooga and colleagues (2012) identified personal stressors and emotional and geographical isolation as pertinent factors in the onset of CSA (*N* = 19, 17 men). The variation in motivation among these studies reflects the contemporary state of the sexual offending literature acknowledging the heterogeneity among those who sexually offend (Smallbone & McKillop, 2016).

A burgeoning body of knowledge has subsequently been generated about schooling contexts specifically, which has more recently included women. Female abusers in schools are slightly younger than their male counterparts, ranging from 30-35 years (CCCP, 2019; Christensen & Darling, 2020; Steely & Ten Bensel, 2020) versus 34.5–42 years (CCCP, 2019; Christensen & Darling, 2020). Women less often engage in penetrative abuse (Christensen & Darling, 2020) and child sexual abuse material offenses (CCCP, 2019) than men. Nonetheless, female school employees do engage in serious sexual crimes against children (Steely & Ten Bensel, 2020). Evidence also suggests that male teachers more often engage in one-off incidents than females (Christensen & Darling, 2020).

The evidence base indicates that women typically offend against older students in mid-late adolescence than men do (Christensen & Darling, 2020; Ratliff & Watson, 2014). Both female and male teachers typically perpetrate against victims of the opposite sex, but women seem more likely than men to abuse same-sex victims (24% vs. 5%) (Christensen & Darling, 2020). Findings vary, but perpetrators typically have one victim. Between half and three-quarters of male perpetrators in schools offend against one victim (CCCP, 2019; Jaffe et al., 2013; Moulden et al., 2010), though can be as high as 90% (Christensen & Darling, 2020). Women especially tend to victimize sole rather than multiple students (CCCP, 2019; Christensen & Darling, 2020).

Qualitative analyses have shed more light on relevant personal and contextual factors, especially for women. These studies either capture various types of institutions but predominantly comprise women in school employees and settings (Darling & Antonopoulos, 2013; Darling et al., 2018; Darling & Hackett, 2020) or focus exclusively on school settings (Christensen & Darling, 2020; Steely & Ten Bensel, 2020). All these studies implicate life stressors and negative emotional states in the onset of women’s institutional CSA. Emotional and sexual gratification have been identified as key motivators, even in the absence of sexual attraction to children (Christensen & Darling, 2020; Darling & Antonopoulos, 2013; Darling et al., 2018). By comparison, 99% of male respondents in Moulden and colleagues’ (2010) sample described their offenses as sexually motivated.

Like those examining male-perpetrated cases (CCCP, 2019; Jaffe et al., 2013), studies report precursor connection, contact, and cumulative interactions between female perpetrators and victims in schools. Examples include spending excessive and unnecessary time together, ‘special attention’, social media contact, and meeting and socializing outside of school (Christensen & Darling, 2020; Darling et al., 2018; Darling & Hackett, 2020; Steely & Ten Bensel, 2020). Steely and Ten Bensel (2020) classified 63% (22/35) of their female sample as exhibiting predatory and grooming characteristics throughout the onset and duration of CSA (e.g., affording special attention to the student, provision of gifts). Darling and colleagues (2018) also identified behaviors that could constitute grooming strategies in 77% of 71 female-perpetrated cases (e.g., meeting outside of school hours, social media contact). However, the authors noted that limitations of the data meant it was difficult to ascertain whether these behaviors were purposively undertaken to commit abuse or whether they represent a progressive unfolding of ‘closeness’ that lacked premeditation. This latter scenario parallels Wurtele’s (2012) ‘slippery slope’ to offending, culminating after a cumulative pattern of boundary transgressions and overly familiar relating, often in the absence of an intention to offend. A lack of appropriate boundaries with children has also been reported in non-institutional samples of female sexual offenders (DeCou et al., 2015).

Christensen and Darling (2020) conducted a comparative analysis of female (*N* = 20) and male (*N* = 20) educators in the U.K and constructed a typology based on available data for all the women and 17 of the men. Seven women and 10 men denied being sexually motivated and minimized their actions; six women and four men experienced poor mental health or significant life stressors preceding their offending and were insightful and remorseful; four women and three men were inexperienced, newly qualified teachers; and three women reported being ‘overpowered’ by their adolescent victims. These authors concluded there were more similarities than differences in the characteristics, motivations, and offense patterns of female and male perpetrators. Moreover, they observed a lack of sexually deviant predisposition in abusive teachers and emphasized the role of situational and contextual factors in school-based CSA.

The extant literature indicates that there are certain opportunities available within school contexts that can prompt CSA (Christensen & Darling, 2020; Darling & Hackett, 2020). Caregiving roles can engender attachment formation that prompts and provokes sexual arousal and/or feelings of emotional closeness and intimacy toward a child (Smallbone et al., 2008; Smallbone & McKillop, 2016). Such roles—like tutoring and counselling—abound for female employees (Darling & Hackett, 2020). Indeed, situational determinants like this have consistently been implicated in the perpetration—and prevention—of institutional CSA (Erooga et al., 2012; Smallbone et al., 2008; Smallbone & McKillop, 2016; Wurtele et al., 2019). Despite the recent advancements of focusing on women in school settings, none have systematically explored the intersection of the personal, situational, and contextual factors unique to school environments.

## Present Study

This study provides a rich, in-depth interpretative analysis elucidating the processes underlying female-perpetrated CSA of adolescents in Australian educational settings. We focused on the age group of 13–17 years because, as outlined in the introduction, this is typically the riskiest developmental period for institutional CSA; it largely neatly aligns with the phase of secondary school and therefore complements an environmental criminological approach of considering secondary schooling contexts as distinct from primary schools; and to remove the presence of pedophilia (sexual attraction to prepubescent children typically operationalized as < 12-year-old) as a confounding variable (which is a qualitatively distinct condition well beyond this study’s scope). For context, seven of eight Australian jurisdictions specifically criminalize the sexual abuse of adolescents above their respective ‘age of consent’ (16 or 17 years) up to the age of 18 by people in positions of authority like teachers. It is not currently a standalone criminal offense in the remaining state. Accordingly, we use the language of ‘incident characteristics’ rather than ‘offense characteristics’ as it does not uniformly constitute an offense in Australia.

Our goal is to provide a contextualized account of the phenomenon to better understand how a professional employee-student relationship transgresses to one characterized by abusive sexual activity (that may constitute offending, depending on legislation). We seek to answer the following question:

What are the contextual, situational, and personal factors influencing the onset of female-perpetrated CSA in Australian educational settings?

We triangulate (1) publicly available documents depicting legal cases stemming from criminal and noncriminal systems and processes (regulatory agencies and oversight initiatives such as professional registration authorities) and (2) expert interviews with professionals who have experience with cases of female-perpetrated CSA in educational settings. These data capture cases that might constitute a criminal offense but have often avoided criminal justice involvement. The inclusion of expert interviews is a novel methodological contribution of this study. Institutional research ethics approval was obtained (Ref No: 2019/1021). See Robertson (2023) for more details.

## Methods

### Data Sources and Sample

#### Publicly Available Legal Documents

Between July 2020 and January 2021, various websites were searched for published decisions of criminal and noncriminal cases of established female-perpetrated CSA in schools, including: (a) jurisdictional Civil or State Administrative Tribunals involving decisions about appeals and hearings pertaining to disciplinary matters or employment screening decisions (e.g., https://www.sclqld.org.au/caselaw/QCAT and https://www.caselaw.nsw.gov.au/browse-court/54a634063004de94513d8289); (b) jurisdictional teaching institutes or registration boards containing facts and findings about professional misconduct matters (e.g., https://www.vit.vic.edu.au/conduct/roda and https://www.trb.sa.edu.au/cases); and (c) Lexis Advance®. The first author combed through search results and assessed them against the inclusion criteria. Cases were included if: (a) the involved employee was female; (b) the child/ren involved were current student/s at the same educational institution where the woman was employed at the time of the conduct; (c) the student/s were aged at least 13 years old and not yet 18 years old at the time of the conduct; and (d) some type of contact sexual act or acts occurred (e.g., kissing, oral sex, or penetrative intercourse). Search results were also cross-referenced with cases referred to in each document. Only documents containing sufficient detail for qualitative analytic purposes were included. Those that only summarized the ‘outcome’ (i.e., confirming that some type of sexualized act occurred) rather than the ‘process’ of how that outcome developed were excluded.

A total of 19 documents depicting 18 cases were identified (see Table A1 for descriptives). Two documents represent the same case but describe hearings in a criminal and noncriminal system and therefore contain different types of information; both were included to ensure a thorough analysis. Some documents relate to criminal trials (i.e., the prosecution or sentencing of criminal offenses in various courts), another to a civil tort, others to the challenging of noncriminal employment screening outcomes, and some to disciplinary decisions by teaching regulatory authorities. The types of procedures associated with these matters are subject to stringent legal scrutiny, thus enhancing their validity and reliability (Almond et al., 2017). Though these cases are in the public domain, no identifiable information is included to maintain ethical compliance; documents were instead assigned an identifying letter (A-S).

#### Expert Interviews

Experts possess specialized knowledge based on practical experience within their respective professional contexts and can be considered “informants” about the phenomenon under investigation (Bogner et al., 2018, p. 659). Experts recruited for interview had professional experience with cases involving the verified occurrence of contact sexual act or acts (e.g., kissing, oral sex, or penetrative intercourse) between female school employees and adolescent students, irrespective of whether it proceeded through the criminal justice system. Both convenience and snowball sampling techniques were employed to identify relevant participants (Petintseva et al., 2020). The kinds of experts contacted included those specializing in (a) workplace investigations of a child protection nature; (b) child safeguarding; (c) ‘reportable conduct’ systems and processes (see e.g. https://ocg.nsw.gov.au/organisations/reportable-conduct-scheme); and (d) teacher regulation (see Table A2 for descriptives).

Semi-structured telephone interviews guided by an interview schedule were conducted with eight experts from three different Australian jurisdictions. Rather than relying on the contentious strategy of saturation (Braun & Clarke, 2021), the final sample was deemed sufficient based on the depth and quality of information obtained (Malterud et al., 2016). Namely, the final sample comprised a niche group of participants with extensive, highly specialized, and relevant professional knowledge about this phenomenon. They had an average of almost 14 years’ experience and had been involved in 1–15+ cases of serious female-perpetrated abuse. Their qualifications included topics such as social work, social science, law, and child protection investigations. These interviews represent a collective experience and expertise that bolsters the results.

Interview questions focused on participants’ knowledge, insights, opinions, and experiences of CSA cases in schools. Topics included establishing experts’ professional background and experience, the case characteristics of matters in which they had been professionally involved, any observed gender differences, agency responses, and prevention. Exemplar questions relevant to this study’s research question include: “I would now like us to discuss some of the female cases you’ve come across. Talk me through a matter you have in mind and describe in detail how it unfolded and developed from its beginning through to its end”; “How do those recollections of specific cases compare to your overall experience with these types of matters? Are they fairly typical of how these cases develop or was there something extraordinary about the cases you mentioned?”; “What are the types of interactions and situations common to the development of sexual acts between employees and students?”; “When thinking about what we’ve discussed so far, how do the female-perpetrated cases compare to the male-perpetrated cases you’ve worked with?”.

Interviewees typically provided descriptive (non-identifying) summaries of some specific cases and drew on their overall professional experience to speak more broadly about the patterns and characteristics they had observed. This conversational and discursive process permitted in-depth and lengthy exploration of topics and a more nuanced insight into the qualities of women’s perpetration. Most experts referenced historical and contemporary (mid-late 2010s and 2020s) cases to draw on the totality of their experiences. Each interview lasted an average of 103 minutes (Range: 70–179 minutes). Recordings were professionally transcribed verbatim, and participants were allocated pseudonyms.

### A Note About the Data Sources

Few women are processed by the Australian criminal justice system (CJS) for sexual offenses (Australian Bureau of Statistics, 2022). Overall, the population (women) and phenomenon subject of this study are incredibly difficult to access in Australia. Thus, both our data sources include information about abusive cases that have not necessarily involved a CJS response. Expert interviews are especially valuable when researching topics involving hard-to-reach populations (Bogner et al., 2018). They are used in criminology generally (Petintseva et al., 2020) and have been used to canvass various professionals’ perspectives of the offense-related motivations of women who perpetrate child sex offenses in different settings (Christensen, 2021). Our application is the first to focus exclusively on educational settings and offers a novel perspective of an untapped resource about an under-studied issue.

The documents were primarily designed for recording information about legal decision-making within the parameters of the respective systems and processes they were drawn from. Details of cases in these documents were typically presented in a straightforward, factual manner, though mitigating circumstances were often more detailed. Some legal documents (especially those drawn from criminal systems and processes) contained information from forensic professionals providing their expert opinion on cases such as assessments of sexual deviance and hypersexuality. The interviews allowed additional in-depth exploration of topics (e.g., gender stereotypes) and more nuanced insight into case characteristics and factors underpinning perpetration. These experts’ opinions and comments were based on all information gathered in a case, including information from perpetrators and victims where available but they are not forensically qualified experts.

### Analytic Process

The legal documents and expert interviews were integrated in a reflexive thematic analysis to identify, compare, interpret, and report patterned meaning in rich detail within and across both data sources (Braun & Clarke, 2022). Integrating multiple data sources (‘triangulation’) strengthens the rigor of qualitative research with small sample sizes (Petintseva et al., 2020) and increases the accuracy and depth of the analysis (Braun & Clarke, 2022). The analysis was theoretically framed by environmental criminology because of its emphasis on the person-situation interaction (Wortley & Smallbone, 2006).

The recursive six-stage analytic process outlined by Braun and Clarke (2022) was followed. Coding began with a fine-grained approach generating many codes of relevance to the research question; upon later review, some were collapsed. Coding mostly occurred at the semantic and inductive level, capturing surface-level meaning contained in the data (e.g., ‘commitment to safeguarding’ or ‘boundary crossings’). At times, a deductive approach occurred where the data were viewed through an environmental criminological lens (e.g., routine opportunity structures such as ‘one-on-one tutoring’ or different offender typologies including ‘predatory’ or ‘situational’). The authors met throughout analysis to discuss developing ideas and interpretations alongside relevant data excerpts and reach consensus, acting as a ‘quality assurance’ technique consistent with this methodological framework (Braun & Clarke, 2022). NVivo (Version 20) software was used for coding and recording and storing the analytic process.

## Results and Discussion

### Descriptive Overview of Perpetration

#### Perpetrator Characteristics

The legal documents pertained to 18 different women. Fourteen clearly specified the age of women at the time of perpetration, which ranged from 20–49 years old, with nine aged 30 years or older (see Table 1). Interviewees echoed a generally bimodal age distribution, identifying women either in their early to mid-20s or early to mid-30s. These data corroborate the average age range of 30–35 years indicated in international scholarship (Christensen & Darling, 2020; Steely & Ten Bensel, 2020).

**Table 1.** Incident Characteristics of Sampled Legal Documents.

Document	Perpetrator age	Victim gender	Victim age	Location/s mentioned^a^
A	N/A	Female	15	N/A
B	N/A	Male	17	Perpetrator’s residence
C	20	Female	15	Victim’s residence
D (and S)^b^	34	Male	14	Caravan park (on vacation); perpetrator’s residence
E	26	Female	16	N/A
F	36	Male	15	School premises (no further specifics); perpetrator’s residence
G	25	2 x females	13 and 17	School premises (art building); perpetrator’s residence; perpetrator’s car
H	N/A	Female	15	Perpetrator’s residence; school camps; school premises (offices)
I	27	Male	16	Third party residence
J	31	2 x males	14 and 17	Perpetrator’s car; public park; perpetrator’s residence
K	31	Female	16	School premises (costume room)
L	22	Male	15	Perpetrator’s residence
M	49	Male	17	Hotel room
N	36	Male	15	Perpetrator’s residence; beach; victim’s residence; perpetrator’s car; school premises (classroom during class time); third party residence
O	29	Male	14	Perpetrator’s residence; hotel room; perpetrator’s car
P	47	Female	17	Perpetrator’s residence
Q	42	Male	17	Perpetrator’s residence; public park; perpetrator’s car; victim’s residence
R	N/A	Male	17	Perpetrator’s residence; perpetrator’s car; hotel room

^a^Listed according to order of mention in the documents.

^b^These two documents depict the same matter but pertain to different systems and processes (criminal and noncriminal). See Table A1 for further detail.

Neither data source consistently identified women’s level of experience in school settings. Documents A and C referred to first-year teachers while P and R identified women with more than 20 years of service. Both data sources indicated that some women had less experience (approximately 3 years), but many had 5–12 years’ experience, which is relatively consistent with prior literature about female institutional offenders (Darling et al., 2018). Some of the experienced perpetrators held leadership roles (e.g., Head of Department) compounding their level of responsibility to, and authority over, their victims. Most women could therefore not be considered young, inexperienced, and naïve employees.

Apparent across both data sources was a dichotomization of relationship and parental status at the time of perpetration. Women were just as likely to be partnered as they were single or separated, and many had their own children. The overall variation across these characteristics highlights the heterogeneity of this offending population.

#### Victim Characteristics

The legal documents contained information about 20 different victims (see Table 1). Ten (50%) were aged 16 or 17 (usually grades 11–12) at the onset of perpetration, six (30%) were 15 (generally grades 9–10), and four (20%) were 13 or 14 years old (typically grades 7–8). All experts recalled victims from the senior years of high school aged 15 and above. The youngest victims (aged 13–14) were captured by criminal legal documents. The age disparity is likely explained because the discovery of younger victims would be taken more seriously and thus more often successfully investigated and prosecuted at a criminal level. Our data indicate that upper secondary students were most at risk of female perpetration, paralleling comparable samples (e.g., CCCP, 2019).

Of the 20 documented victims, eight (40%) were female and 12 (60%) were male. Interviewees also recalled more cases with male victims, but six each recounted at least one case with a female victim. Female victimization was thus higher than in previous research where girls constituted one-quarter of victims by female teachers (Christensen & Darling, 2020). This difference might be attributable to our broader inclusion criteria capturing *any* schooling employee, not just educators (e.g., coaches, administrative staff).

Six of the 20 victims (30%) described by legal documents were considered especially vulnerable, and four experts also noted this. Such vulnerabilities included: mental health issues; sexual identity concerns; trauma histories; relationship difficulties; life stressors; substance misuse; emotional distress; crisis; poverty; dysfunction; bullying; and learning difficulties. Some of the qualitative data suggested that not all female perpetrators deliberately target and exploit particularly vulnerable victims for sexual abuse (beyond the inherent age and power differentials). Overall, there was no typical victim profile.

#### Incident Characteristics

A range of sexual acts were described in the data. Civil legal documents sometimes included a broader description of behavior than criminal cases that particularized the specifics of offenses. For example, Documents C and K referenced a “sexual relationship” but provided no further detail. Generally, though, the documents described behaviors such as kissing, sexual touching of genitalia, oral sex, digital penetration, and penile-vaginal penetration. Fourteen of 18 cases (77.78%) clearly identified the most serious end of the abuse spectrum involving some form of genital penetration. Experts’ accounts mirrored this continuum and seriousness of sexual acts. Additionally, Kimberly mentioned exposure to child abuse material and exchange of sexually explicit material, and Daniel referred to sexual indecency in the form of masturbation. These acts are consistent with the continuum reported in previous studies (Christensen & Darling, 2020; Steely & Ten Bensel, 2020).

The locations of abusive incidents mentioned in legal documents are presented in Table 1 and are listed according to their order of mention in the documents. Interviewees referenced the following specific places: school grounds (Dylan), perpetrator and victim residence (Kimberly), third party residence (Daniel and Dylan), motel (Kimberly), and school camp (Melissa). Taken together, these locations are consistent with prior literature (Christensen & Darling, 2020; Steely & Ten Bensel, 2020).

### Patterns Preceding Perpetration

The three themes and one subtheme produced from the two data sources are summarized in Table 2. Each theme is detailed below.

**Table 2.** Overview of Generated Themes and Subtheme.

Theme name	Theme (and subtheme) definition
‘There’s something amiss’: Underlying individual susceptibilities	Challenging the conventional stereotype of child sex offenders as sexually motivated deviants, this theme captures the influence of unmet relational, emotional, and intimacy needs in women’s sexual offending. Commonly underpinning these unmet needs is a pattern of life stressors and adverse personal circumstances characterizing perpetrators’ lives prior to perpetration that seemingly increased their susceptibility to offending.
‘Asleep at the wheel’: Permissive institutional cultures	This theme captures how the values, attitudes, and practices (or omissions) characterizing schooling contexts can act to enable female perpetration. Elements such as commitment to safeguarding, embeddedness with local community, degree of religious observation, and dismissal of rumor are influential here. The subtheme of *‘Is it someone being motherly or maternal?’: Giving women the benefit of the doubt* focuses on a particularly pertinent aspect of the broader institutional backdrop and describes the contribution of gender scripts and sexual stereotypes to a school’s culture. This subtheme encapsulates the implicit trust often afforded to women, minimization of their potential harm, and dismissal of same-sex perpetration that serve as (potentially subliminal) tolerance for female-perpetrated wrongdoing.
‘Cultivating a relationship’: Opportunities for cumulative intimacy-promoting situations	This theme reflects the cumulative intimacy-promoting opportunities within schooling environments for situations and interactions conducive to the sexualization of an otherwise professional relationship. Here, the materialization of the ‘slippery slope’ to perpetration in educational settings is illustrated, including the manifestation of boundary crossings and the development of close emotional bonds.

#### ‘There’s Something Amiss’: Underlying Individual Susceptibilities

Female perpetrators were clearly depicted as sexually motivated predators in three legal documents. Document G outlined a woman’s grooming and manipulative behavior and the sentencing judge concluded she “deliberately exploited her position for her own advantage and sexual gratification.” In Document H, sexual gratification was referenced as the “sole motivation” and labelled the perpetrator a “serial sexual abuser”. Sentencing remarks contained in Document Q referred to the perpetrator as “grooming” the victim prior to a “fairly confined” period of offending. These descriptions align with ‘predatory’ offenders marked by an ingrained sexual attraction to children and a purposive grooming process to facilitate sexual abuse (Wortley & Smallbone, 2006).

Interviewees rarely conceived of women in this manner. Indeed, Dylan, Daniel, Kirk, and Claudia all expressed a view that in their experience, a predatory, sexually motivated pattern more often characterized male perpetration. Cynthia was an exception. She thought some women did intend for their cumulative precursor interactions with students to culminate in sex, though noted the fallibility of this reasoning because of the difficulty in establishing underlying motivation and intent. Nonetheless, she remarked, “I can’t see where they’ve [women] tried to stop…they know what’s happening…otherwise it would stop.” Cynthia’s account of persistence portrays a perpetrator both aware of their actions (and subsequent consequences), as well as unaffected by other subjugating factors such as emotions or life stressors. Cynthia also recounted a one-off incident where an employee and student who had little prior interaction “ran into each other” in a social setting and had sex. This scenario is reflective of impulsive sexual gratification and weakened self-control and inhibitions (due to intoxication, e.g.) characterizing ‘opportunistic’ offenders (Wortley & Smallbone, 2006).

In comparison—and highlighting the heterogeneity of this population—a conceptualization of emotionally ‘at risk’ women, ostensibly increasing their susceptibility to offending, was more widespread. For instance, a forensic psychologist in a case adjudicating perpetration against a 14-year-old boy stated that the offender’s motivation was not “that of a sexual predator with the sole intention of sexual gratification” (O). Here, the sentencing judge commented that the offender did not “possess an unhealthy interest in children” but was “vulnerable to having a relationship with a teenage boy.” Other sentencing remarks in criminal cases identified perpetrators as “emotionally vulnerable” (J), in an “emotionally fragile state” (N), or “psychologically vulnerable” (Q). The legal documents generally referred to these women as being a low recidivist risk due to their lack of an ostensibly predatory sexual motivation.

Such remarks about women’s susceptibility may be controversial and perturbing. At first glance, they could be viewed as gendered interpretations of perpetration whereby women’s agency and responsibility are diminished in a way that is absent in the sentencing of male perpetrators. They could therefore be illustrative of the courts’ leniency toward female sexual offenders (Deering & Mellor, 2009) and minimizing and romanticizing media portrayals of court cases (Hayes & Baker, 2014). However, all these women were deemed culpable and served custodial sentences thereby reflecting contemporary findings that gender does not influence sentencing outcomes (Knoche & Russell, 2021). Furthermore, the language around susceptibility did not only appear in the legal documents; many experts also observed a lack of ostensible (initial) underlying sexual intent or deviance and a level of susceptibility in female perpetrators. Claudia commented: “I think some of them are [sexually] motivated, but I think more of them are vulnerable and their [initial] intentions are good.” Dylan concurred:

I think there are times where the teacher may not have set out…with the intention of engaging sexually with the student. There are occasions where the teacher initially seeks to support a student’s wellbeing but that the professional boundaries quickly get blurred and lead to a sexual interaction.

Some experts also identified a similar pattern of susceptibility and boundary slippage preceding *male* perpetration but noted it was especially marked in female-perpetrated cases. Again, the interviewees did not cite this susceptibility to excuse perpetration, or view it as somehow more acceptable and less harmful. Rather, based on their substantial experience, they were observing material differences among offending populations and challenging society’s narrow typecasting of sexual offenders as evil ‘monsters’ to better understand the full range of perpetration and its onset. A level of susceptibility might thus be a fitting portrayal of some offenders, potentially representative of distinct offending patterns and risk factors rather than gendered discourse. This mirrors previous findings that situational factors—rather than predatory predisposition—are highly relevant in the onset of offending for female and male institutional offenders (Christensen & Darling, 2020; Erooga et al., 2012). Experts’ conceptualizations of female (and some male) offenders as ‘well-intentioned yet susceptible’ parallel Wurtele’s (2012) observation of most institutional perpetrators. Namely, that most do not intend to offend but make more poor judgements and “slip down the slippery slope of boundary violations” (p. 2444), especially when emotionally and personally susceptible.

Female susceptibility stemmed from adverse personal circumstances and life events prior to perpetration, which aligns with previous research (Christensen & Darling, 2020; Steely & Ten Bensel, 2020). Circumstances related to *internal* states including personality deficits or psychological conditions and encompassed mental health issues (e.g., depression or anxiety); emotional and relational immaturity; poor self-esteem; drug and/or alcohol abuse; sexual identity issues; and loneliness. Adverse circumstances also related to *external* life stressors comprising events such as relationship stress and difficulty; permanent dissolution of romantic relationships; trauma histories; and grief and loss. At times, these circumstances were complex and often overlapped. Interviewees often expressed women’s susceptibility as a form of *general discontent* permeating their lives:

…things that aren't quite right in their personal lives. There's something missing for them…there's a want for something. (Claudia)

…relatively unsettled in some capacity in their life…not coping with life… (Dylan)

…there's something amiss, there's something not content within their lives…they had stuff going on in their personal lives that perhaps wasn’t ideal, or wasn’t being managed… (Kimberly)

Like the underlying philosophy proposed by the GLM (Ward & Gannon, 2006), most women had unmet emotional and relational needs prior to perpetration that interactions with students appeared to fulfill (whether they were aware of it or not). Some “craved understanding and affection” (N), for instance, or had an “intense desire for intimacy and affection” (S). Deeper relational needs were echoed in experts’ accounts too. For example, Cynthia, commented, “…it’s sort of like they’re trying to form a boyfriend/girlfriend relationship.” Melissa reiterated this by recalling a particular perpetrator who viewed the victim as her “boyfriend” and treated him as such. Dylan noted that in some instances, the victimization of girls involved women who expressed some “confusion about their own sexual identity” and were seeking to reach resolution on this matter. Previous studies have also observed female perpetrators questioning their sexual identity (Darling & Antonopoulos, 2013; Darling et al., 2018), violating emotional boundaries by elevating the student to equal adult status (Wurtele, 2012). Claudia also connected these unmet needs and the tendency for older students to be victimized because they allow an:

…ability to have more than a physical connection. This is where that emotional connection comes in and this getting something from the relationship, not necessarily in a sexual way, that it's this want. It's harder to fill that…with the young students.

Both data sources provided evidence of relational enmeshment where the lives of both parties became intertwined. Declarations of being “in love”; cohabitation; and willingness to jeopardize employment, marriages, and family life by pursuing the ‘relationship’ with the victim were common. These examples are consistent with the extant literature (Christensen & Darling, 2020; Darling & Antonopoulos, 2013). Such enmeshed relationships can result in a high degree of entanglement, which may be where potential harm lies, particularly for very vulnerable victims. For example, the impact on a victim who, upon detection of the abuse, is developmentally ill-equipped to deal with, or understand, the need for disentanglement from each other’s lives after intense enmeshment. Document N’s sentencing remarks reflect this sentiment: “…the appellant’s offending consisted of…taking over and dominating his life…a graver form of offending than would be the commission of individual sexual offense of the kind more often encountered.”

Both data sources indicate that women’s offending was typically considered a serious departure from their usual exemplary behavior. As Claudia remarked, “really good people can make really bad decisions.” Her comment reflects what Smallbone (2016) termed the “disturbing ordinariness of many, if not most, sexual offenders” (p. (2) whereby regular people devoid of deviancy can offend in certain circumstances. This theme captures the widespread observation of ‘situational’ offending, where otherwise prosocial individuals without an entrenched sexual attraction to children offend in reaction to environmental circumstances (Wortley & Smallbone, 2006).

#### ‘Asleep at the Wheel’: Permissive institutional cultures

Institutional culture comprises “the collective values and practices that guide the attitudes and behavior of staff and volunteers”, conveying—directly and indirectly—“the way things are done” in a workplace (Royal Commission into Institutional Responses to Child Sexual Abuse, 2017a, p. 146). Both data sources contained explicit and implicit references to institutional culture. One related to the conscious and intentional effort required to create a robust safeguarding culture. Educational environments that relied on a passive, assumed culture (e.g., employees’ ‘common sense’) seemed more at risk of enabling perpetration than those that actively created, expressed, and actioned a commitment to safeguarding. Multiple interviewees emphasized how it is incumbent upon leaders to intentionally create a culture that genuinely values safeguarding rather than simply viewing it as legislative compliance; where the values are *embodied* and not just purported. Tracy concluded that culture is “everything” and that it “certainly is a massive [preventative] factor”.

A school’s commitment to a safeguarding culture was connected to the contextual concepts of time and place. For example, some participants recounted historical allegations from a time when far less regulation existed. This meant the accepted ‘way things were done’ were riskier (e.g., consuming alcohol with students) because, as Melissa noted, “there was not the same awareness of the risks 30 years ago as there is now.” The resultant advances in understandings of CSA dynamics have updated practices and reduced offending opportunities. Conversely, features of modernity, such as the advent of technology, were identified as posing their own set of challenges for contemporary schools to navigate.

The concept of place was expressed in multiple ways. One example was faith-based institutions as a specific place with a unique ‘way of doing things.’ Document H described the culture of an insular orthodox religious school where serious female-perpetrated abuse occurred. It noted that the employee’s responsibilities and relating with students “concerned the very core of their beliefs, values and everyday living...the community is bound together in an incredibly tight, enveloping way…” Here, the school was a conduit for religious transmission and education such that they essentially blended to become one entity. Both Melissa and Tracy spoke about religious schools that had historically operational organized CSA with multiple predatory offenders. Melissa commented that “people were asleep at the wheel” in these places and female perpetration consequently went unnoticed.

Multiple interviewees also noted that faith-based settings can engender dual roles and employee-student relationships because of the crossover between religious and schooling life (e.g., mutual attendance at church activities). This overlap not only provides additional opportunities for contact and rapport-building with children (thus facilitating subsequent abuse), but also blurs the boundaries of what is deemed acceptable professional behavior across different settings. Cynthia, for instance, recalled a perpetrator driving a child (who later became her victim) home from church, a practice that would have been questioned in the educational context, but at which “no one in the church really blinked an eye.”

The second intersection of institutional culture and place was ‘embeddedness with the local community’. Problematic crossover between schooling operations and other aspects of ordinary life were frequently found across both data sources. Namely, mutual extra-curricular, sporting, and personal activities (e.g., friendships with parents of students) often preceded perpetration. Claudia remarked this “allows for these personal relationships to occur alongside the professional relationship when in fact it should be professional the whole time.” This was especially common in regional locations, where Dylan commented that teachers are “so ingrained in the community” they are expected to have additional involvement in the community beyond their professional role and “they’re often involved in each other’s lives beyond the confines of the school premises.” Both Dylan and Daniel also mentioned that employees and students being in each other’s homes was not uncommon in regional settings. These scenarios create increased opportunities for contact outside of the formal schooling setting and promote porous boundaries that can erode a professional mindset. Documents D and *N*, for instance, depicted women’s perpetration initiating through the peer-aged relationships between their own children and the eventual victim, where offending ultimately occurred during sleepovers.

Failures to promptly report and respond to early concerns were crucial aspects of a school’s safeguarding culture featuring across both data sources. The occurrence of known, yet dismissed, rumor circulating before the eventual discovery of sexualized conduct was frequently conveyed across the data and is congruent with prior studies (e.g., Darling & Hackett, 2020). Barriers, or “blind spots” (Kirk), to reporting rumors or early indicators included employees who were: parents and had received information informally from their children; former students who had heard about information when enrolled; or friends with the alleged perpetrator. Concerns about reporting falsehoods and the serious repercussions also curtailed timely reporting. Mishandling of known rumors also extended to leadership. Tracy voiced the need for leadership to have “zero tolerance” for inappropriate behavior. Otherwise, any expressions of commitment to safeguarding are superseded by unaddressed transgressions which implicitly convey that wrongdoing, is, in practice, tolerated.

##### ‘Is it Someone Being Motherly or Maternal?’: Giving Women the Benefit of the Doubt

Given its irrelevance to legal proceedings, commentary about the role of existing gender scripts and powerful sexuality stereotypes on institutional culture was not apparent in the legal documents. This topic was therefore only evidenced by the expert interviews. Although they acknowledged vast improvements over the years, interviewees believed schooling personnel (regardless of role) remained susceptible to stereotypical fallacies. As Kirk remarked, “it’s difficult for most people to conceive of a woman as the perpetrator”, which is consistent with traditional sexual scripts of women as asexual and passive recipients of sex (Denov, 2003; Saradjian, 2010). Participants expressed how inappropriate female-perpetrated behavior can therefore be allowed to continue for a longer duration, and thus escalate to sexual abuse, than similar behaviors perpetrated by men because:

…there can be this trust [of women]…[and an assumption that] females don’t do that. A little bit of a double standard... (Claudia)

I think people are more inclined to view females as more caring and pastoral and allow more latitude... (Melissa)

Participants acknowledged the gendered nature of sexual violence, and thought the overrepresentation of men as perpetrators could influence delayed detection and reporting of women. However, Kimberly explained that witnesses engaged in an additional level of ‘filtering’ and meaning-making when processing women’s transgressions:

[Witnesses in male-perpetrated cases] are more comfortable in telling you what they see, not how they're interpreting it. There's still a level of people trying to interpret female behavior; it's not clear…there is no sort of question around whether that is caring or nurturing [when] it's a male…There's still a…confusion around “is it someone being motherly or maternal towards the child and it's been misinterpreted?” when it involves a female.

Overall, women seemingly continue to be deemed more trustworthy than men and afforded the benefit of the doubt if they transgress. This inadvertently condones inappropriate behavior that escalates to sexual abuse if not disrupted (Darling & Hackett, 2020).

In keeping with enduring stereotypes that minimize the impact of female-perpetrated CSA (Denov, 2003; Russell & Gruys, 2022), multiple experts spoke of a lingering tendency to underestimate the harm women can cause:

…I think community perceptions are that if a male student has a relationship with a female staff member, it's not as bad for the student. It's almost like a “good on you, mate, that's great”…(Claudia)

I think there's an erroneous view as well, that, if they're [victims] abused by a female, it's less damaging than if they're abused by a male. (Cynthia)

Thus, female perpetration would be viewed as *ineffectual, benign,* or even *welcomed* and may not provoke the same urgency to be reported as male-perpetrated abuse. Multiple interviewees challenged the minimization of women’s perpetration and remarked on the profoundly negative impact male victims reported to them.

The dominance of heteronormative sexual scripts also influenced safeguarding culture. Some experts indicated that schools reduced interactions between women and boys, whilst simultaneously dismissing those between women and girls. Daniel recalled an example of schools allowing one-on-one tutoring between female employees and female students to the exclusion of male students. This is problematic given the high numbers of female victims portrayed in our data. Overall, female perpetration can be subliminally tolerated when traditional gender scripts remain unchallenged within a school’s cultural landscape (Darling & Hackett, 2020).

#### ‘Cultivating a Relationship’: Opportunities for Cumulative Intimacy-Promoting Situations

**Figure 1.**
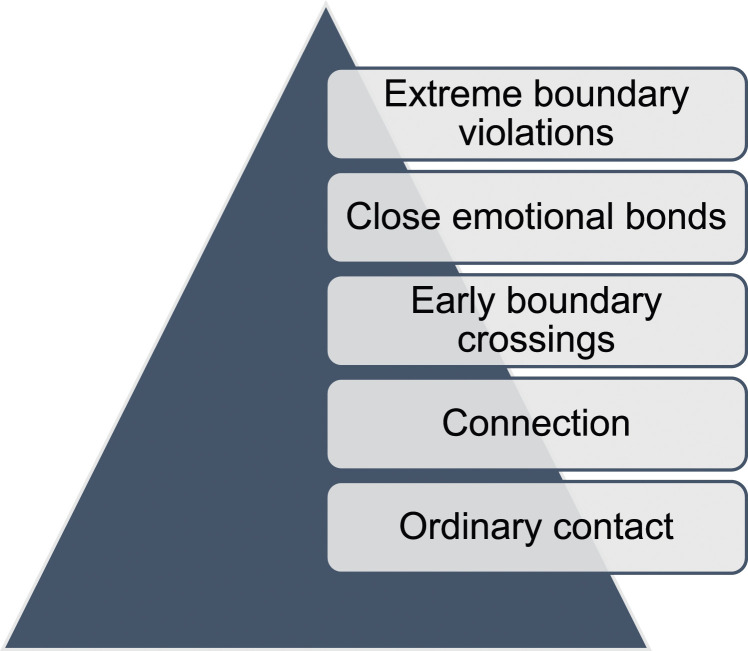
Overview of process preceding most cases.

As will be evidenced, most cases involved a series of interactions between perpetrators and victims prior to perpetration that were borne from the immediate schooling environment. Dylan described the process of female-perpetrated cases as more often involving women “cultivating a [non-sexual] relationship that may turn sexual over time”. Such cultivation involved a gradual erosion of professional boundaries that define the parameters of relating between staff and students (Wurtele, 2012), beginning with cumulative lower-level, incremental behaviors preceding sexualization (Figure 1). What begins as ordinary contact routinely available in schools, leads to a connection being formed. From here, early professional boundary crossings arise, close emotional bonds are developed, and extreme boundary violations occur.

Cases across both data sources tended to initially involve interactions based specifically on legitimate *educational* (two documents and four interviewees) or *welfare* (eight documents and seven interviewees) purposes, mirroring prior research (Darling & Hackett, 2020; Steely & Ten Bensel, 2020). Though it is possible, interactions were not necessarily intentionally orchestrated by employees for deviant reasons, rather, they were an expected by-product of their customary roles. Kimberly explained that, in her experience, cases “commence[d] from something that is within the norm of the expectation of a teacher” and something employees believed “they had grounds to do.”

Learning support was typically provided as one-on-one tutoring thereby allowing increased time spent together alone. This often occurred in unsanctioned locations (e.g., residential settings) outside of usual school hours thus limiting oversight and scrutiny. Formal or informal welfare roles can promote intimacy if undertaken carelessly and can be exploited by those with deviant motivations. Such roles facilitate additional time spent alone and unsupervised and can include discussions of personal and intimate topics elevating the employee to trusted confidante. Cynthia and Kirk commented that the usual boundaries of employee-student relating “blur” in either of these scenarios. Women may be especially at risk of participation in these roles—either of their own volition or by management’s direction—given the aforementioned stereotypes. Cynthia observed that the higher victimization of older students is explained by these educational and welfare practices that intrinsically more often cater to the pressures of senior schooling.

Experts explained that opportunities for one-on-one interactions fostered the development of trust, comfort, familiarity, closeness, and, ultimately, a sense of connection over time. Claudia remarked that “it’s the ability for the initial connection to be established” without scrutiny that forms the basis of a professional relationship progressing to sexualization. An “innocuous conversation” about a shared mutual common interest—such as sport or entertainment—was the typical instigator of sexualized relationships (Dylan). Some experts spoke to women’s susceptibilities serving as a point of connection with victims who were grappling with similar issues (e.g., mental health or sexual identity). Though the intention of assisting students would likely be endorsed by leadership, the resultant level of connection and entanglement in each other’s lives would not. A high degree of embeddedness with the local community and overlapping dual relationships also expedited the formation of trust, familiarity, and discovery of common interests.

This employee-student connection gives rise to a climate promoting initial professional boundary transgressions and poor professional judgement, which is typical of situational rather than predatory types of sexual wrongdoing (Christopher, 2017). Both data sources described numerous types of boundary crossings at this early stage, including: one-on-one transportation (13 documents and four interviewees); spending increasing amounts of time together during the school day (e.g., recess and lunch breaks, arriving at or leaving school simultaneously) (nine documents and eight interviewees); socializing outside of school (sometimes including the consumption of alcohol) (12 documents and seven interviewees); home visits (16 documents and seven interviewees); increased levels of one-on-one communication (including social media contact) (12 documents and six interviewees) and discussion of personal topics like relationship difficulties (12 documents and seven interviewees); gift giving (eight documents and two interviewees); and physical contact (e.g., hugs and massages) (12 documents and five interviewees). Overwhelmingly, these types of breaches were unnecessary for the role and unauthorized in the workplace setting, which Document M illustrates:

[Employee] attempted to counsel the student when she was not authorised to do so, and she failed to notify his parents, guardian, or supervisory staff about those welfare concerns. This occurred in the face of having been directly questioned about the student’s behaviour…[she] did not have any approval to privately meet with the student…[and] did not report the meeting to any person.

Multiple preliminary boundary crossings typically overlapped across numerous and different settings, again indicating the pervasive and enmeshed nature of women’s general involvement in victim’s lives. Early boundary crossings lacked openness and transparency making them particularly high-risk practices in the workplace setting. Claudia commented that in these cases where the parameters and boundaries of relating “blur” and “erode”, employees “are absolutely at risk of harming a child.” Emotional intimacy developed in the context of these gradual boundary crossings and preceded physical contact.

Both data sources indicate that early indicators of escalating risk were detected where close bonds had ostensibly developed. Kimberly described a case where perpetrator-victim interactions “looked unusual.” The relations between perpetrator and victim in these cases *look* different, generate *unease*, and *stand out*. In some cases across both data sources, they reflected juvenile peer-like friendships involving excessive time spent together and the use of nicknames (six documents and four interviewees). Other interactions were noticeably ‘flirtatious’ in nature (two documents and three interviewees) and involved unnecessary physical contact. Yet others ‘looked’ and ‘felt’ like romantic ‘relationships’:

[Witness] noticed them at a school event and walked past the young student and thought he must have a new girlfriend. [Witness] looked back and saw that it was the teacher, and it made her feel uncomfortable, and it caught her attention to the point that she felt the physical closeness and the mood around them was outside the student-teacher relationship. (Kimberly)

[Colleague] observed the student waiting outside the classroom “nearly every day, quite a lot of lunchtimes and sometimes after school.” (Document K)

…there was suspicion amongst some staff that “something was going on” as the teacher’s behaviour included flirting with the boys, and engaging in juvenile behaviour with the students, such as water fights. (Document B)

Without disruption, these close emotional bonds ultimately led to extreme boundary violations including ‘sexting’; mutual drug use; sleepovers; and cohabitation.

This incremental process parallels Wurtele’s (2012) notion of sliding down “the slippery slope of boundary violations” (p. 2444). Indeed, three interviewees explicitly referred to this concept when recalling how cases typically unfolded in their overall experience:

…transitioning from appropriate interactions into this rapid decline down the professional boundaries slope…whatever the intention is…it just spirals… (Claudia)

…something that's essentially innocuous at the start can…put a teacher very quickly on a very slippery slope that's hard to get out of. (Dylan)

It's a very slippery slope that occurs for a teacher who may not have any intention at the start of developing a relationship with students in their care…(Tracy)

## Summary and Implications

To our knowledge, our study represents the first examination of women’s sexual perpetration in Australian educational settings (especially secondary settings). We found that perpetrator susceptibilities, multidimensional institutional cultures, and intimacy-promoting situations coalesced to create high-risk conditions for female school employees. This reiterates the utility of applying environmental criminology to the problem of institutional CSA to understand the person-situation interaction in proximal environments (Wortley & Smallbone, 2006).

In terms of personal factors, unmet relational and emotional needs and adverse life circumstances and stressors were underscored as amplifying women’s susceptibility to perpetration. Meeting basic human needs via sexual offending aligns with previous findings in educational institutions (e.g., Christensen & Darling, 2020; Steely & Ten Bensel, 2020) and the GLM rehabilitation model (Ward & Gannon, 2006). These proximal individual risk factors could potentially be identified and mitigated within workplaces. The ‘ordinariness’ of women was, however, apparent overall, suggesting that most effective prevention efforts will target elements of the broader environment.

The influence of the broader institutional backdrop was also emphasized. Weak safeguarding cultures reflecting passivity, high degree of religious observation and embeddedness with the local community, and dismissal of rumor all influenced female perpetration in educational contexts. This finding is again consistent with previous literature (e.g., Darling & Hackett, 2020). These aspects of a school’s culture therefore require explicit and intentional reflection, scrutiny, and ongoing review.

The qualitative analysis revealed enduring sexuality stereotypes (e.g., Denov, 2003) as an especially powerful aspect of the broader institutional environment that uniquely enable contemporary female perpetration. The continued inability to conceptualize women as sexual offenders and the subsequent implicit trust afforded to them can act as (subliminal) permission for, and tolerance of, transgressions and wrongdoing in the workplace (Darling & Hackett, 2020) in a way that is not apparent for men. Our results suggest that schools—and indeed, broader society—still have a way to go in combatting this blind spot to protect children from harm.

Finally, the types of one-on-one opportunities within school environments that enable the ‘slippery slope’ (Wurtele, 2012) into close, enmeshed relationships were illustrated. Risk factors included excessive and unnecessary time spent together, socializing outside of the school settings, unwarranted communication, and a level of closeness and overly familiar interactions beyond what a typical employee-student relationship resembles. These results mirror previous examinations of women’s institutional offending (Darling & Hackett, 2020; Steely & Ten Bensel, 2020). The results suggest that a blasé approach to the management of employee-student interactions that lacks thoughtful intentionality can engender risk in school contexts. Moreover, recognizing and reporting overly familiar connections provides schools the chance to disrupt these dynamics before they escalate (or continue). All of this is not to say that a sexual desire and motivation was non-existent at the time of women’s perpetration. It is merely to highlight the heterogeneity of this population and that not all fulfil the inherently deviant and pathological typecast of those who perpetrate child sex offenses. Intimacy-promoting situations are not only problematic because they can be used to facilitate intended and planned offending, but because they can engender sexual motivations for the first time (Smallbone et al., 2008). The likelihood of this developing increases with an employee’s overall susceptibility, including experiencing negative emotional states due to acute personal problems (Marshall et al., 2006; Wortley & Smallbone, 2014). In this way, sexual motivation for many of these women can be viewed as a byproduct of intimacy formation developed through cumulative interactions rather than the impetus for them.

### Lessons for Prevention

Experts identified some similarities between female and male perpetration, including underlying emotional and relational needs and the ‘slippery slope’ to perpetration. Thus, the following suggestions are considered broadly prudent practice in schools that may extend beyond women alone. Moreover, given our sample focused on abuse against adolescents, these recommendations are most applicable to secondary schools. We focus on creating safer schooling environments and encouraging reflexivity at the individual employee level.

Schooling leadership should proactively audit their current operations and clearly prohibit and disrupt the following practice: one-on-one transportation, tutoring, and welfare support; social media contact; and socializing outside the workplace (in public and residential settings). Maintaining professionalism within and external to the school should be emphasized while proscribing the development of overly familiar relationships that appear peer-like, flirtatious, or romantic. Exceptions to the rules need to occur with the school’s consent and risk assessment to maximize transparency. Suitably qualified staff (e.g., school counsellors) may work one-on-one with adequate transparency and oversight. Other employees like teachers, however, should not be providing intensive support to students by themselves. These rules also need to apply to same sex (i.e., female-female) interactions.

Staff need to be trained on—and have explicit conversations about—employee sexual misconduct and the application of professional boundaries (Wurtele et al., 2019). These sessions could include awareness raising about female-perpetrated abuse in schools, especially against senior school students. Staff should be encouraged to reflect upon the socially conditioned gender stereotypes underscored in expert interviews to ensure female-perpetrated misconduct is not minimized or ignored. Training ought also to include content on the ‘slippery slope’ to perpetration involving indicators of overly familiar or unnecessary emotional connections. Those who cannot follow the rules or apply boundaries appropriately in child-related employment require clear directions, advanced training, and firm disciplinary consequences. A school’s rules and training (a) guides employee’s own behavior and (b) enhances their contextual awareness (Reynald, 2010) of their workplace, thereby bolstering their capacity to act as protective guardians of children.

To disrupt early boundary crossings from escalating, schools should require—and encourage—reports of lower-level concerning conduct that breaches policy, procedures, and expectations. This will likely yield more confident identification from reporters than having to identify an early stage of grooming, which requires knowledge of an underlying sexual intent. Indeed, in Scurich et al.’s (2023) study, roughly 59% of participants in two studies (*N* = 371 and 79 respectively) deemed precursor behaviors (e.g., hugging, socializing) as concerning and/or inappropriate. Though most participants did not definitively affirm them as grooming, they *did* recognize them as problematic. This rate would likely increase when surveying only institutional stakeholders with contextualized knowledge of the schooling setting and institutional policies. To aid their knowledge and reporting, documented rules about employee conduct should be disseminated to key stakeholders who can act as protective guardians, including staff, family, and students (Robertson et al., 2024). Age-appropriate versions of such policy documents should be adapted for students and can be complemented with brief information sessions about appropriate employee conduct, irrespective of gender (Robertson et al., 2023). Further research about the effective training of stakeholders to recognize and report early indicators is required.

We endorse Christensen and Darling’s (2020) call for schools to support susceptible employees who may be experiencing hardship to ensure their emotional and relational needs are met in healthy, age-appropriate ways. Various options can be considered once such an employee has been identified, including referrals to available internal counselling services; Employee Assistance Programs; and external psychological services for schools with sufficient financial resources. This should occur as early and as often as possible. Where feasible, management can encourage employees to take a period of leave at times of known stressors (e.g., relationship breakdown or grief and loss). Schools should create a supportive culture and encourage help-seeking behavior in their employees (Darling & Hackett, 2020).

Finally, schools should encourage their employees to regularly engage in emotionally mature, self-aware, and reflexive practice embedded into everyday operations. Due regard for professional boundaries, combined with reflective and intentional practice that promotes openness and transparency, is essential to preventing CSA in schools (Christopher, 2017; Wurtele, 2012). Without these, ordinary contact can quickly transcend to excessive familiarity for employees. Staff should be prompted to regularly reflect and self-evaluate their conduct in keeping with expectations to self-regulate and adjust their behaviors, if required. This can be achieved through prominently placed posters displaying reflective questions in staff rooms, offices, bathrooms, or other spaces where staff congregate regularly. Examples of questions to display include:

• Am I treating a student differently to the way I treat others?
• Are we spending a lot of time together alone?
• Are we socializing outside of school?
• Do we discuss personal matters?
• Am I counselling them?
• Do we communicate outside of school?
• How do I behave around them?
• Do I think of them as a friend?
• Do I have feelings for them?
• Would I be comfortable if my employer, colleagues, or a parent saw our interactions, or do I need to modify my behavior?
• Am I currently struggling and need some support?

The posters should also include a statement encouraging staff to consult their superior if they need clarification on maintaining and applying professional boundaries. These questions can also be appended to formal protocols like Codes of Conduct.

Such reflexive practice can also occur relationally with open dialogue between staff holding each other accountable for upholding institutional standards and expectations. For instance, noticing to a colleague when they have engaged in conduct that is against school rules or could easily be open to misinterpretation. We note however that such conversations should not replace internal reporting obligations.

## Limitations and Future Research Directions

The qualitative data sources allowed for detailed and in-depth insights into factors influencing the onset of women’s perpetration against adolescents in Australian schools. The overall sample, however, was relatively small despite triangulation and its qualitative nature limits generalizability to other populations. Some legal documents contained information from perpetrators and psychological or psychiatric assessments from qualified forensic professionals, but they were not primarily designed for research purposes. Our interviewed experts were not forensic specialists, and we did not seek information directly from perpetrators. Though our results make a valuable contribution to the overall state of the literature, these limitations necessitate interpretive caution.

Accessing legal documents about more contemporary cases would have been useful. Almost three-quarters (72.22%) of documented legal cases in this study occurred between 2000–2010 (13 of the 18; see Table A1). Substantial delays in reporting institutional CSA occur (Royal Commission, 2017b), and the subsequent investigations, hearings, and publishing of outcomes (if at all) can be lengthy. The public availability of recent cases is thus limited. The sampled period does not impact certain features of the results (e.g., personal circumstances of perpetrators). Safeguarding within educational institutions has however changed since that decade and more recent examples of institutional culture and high-risk practices would have been valuable. The expert interviews offset this limitation to an extent as most moved between referencing historical and more contemporary cases. This facilitated analysis about changes over time and offered valuable insights into the operations of contemporary schools based on the experts’ current involvement with them.

Beyond replicating the study with a larger sample, our understanding of this phenomenon can be further advanced by conducting interviews with known perpetrators (irrespective of formal criminal justice involvement). There are limitations of offender-based research in terms of the reliability of self-report data and limited recall (Bernasco, 2011). Nonetheless, interviews could probe preceding motivation and intentions, how perpetration unfolded, and what would have effectively prevented it (Jacques & Bonomo, 2017). These details—and their temporal ordering—could make valuable contributions to the totality of the knowledge base about contemporary cases, especially when combined with other collateral sources of information such as victim perspectives. Interviewing both women and men to further reveal similarities and differences in their offending profiles would be especially worthwhile.

## Conclusion

Our study supports previous theorizing that many institutional perpetrators develop offense-related motivations for the first time during their professional employment rather than having a predisposed or deep-seated sexual attraction to children that compels them to seek child-related employment for the purposes of abuse. The key to prevention lies in strengthening the environments in which employees operate. Prevention of female-perpetrated CSA in schools is possible with the creation of reflexive environments where the potential for women’s sexual perpetration and employee-student power differentials are recognized, and professional boundaries are maintained and enforced.

## Appendix

Table A1 and A2

**Table A1.** Characteristics of Sampled Publicly Available Documents.

Document	Jurisdiction	System	Description of case^a^
A	Vic	Civil	Disciplinary decision about suitability of ongoing teacher registration (2003)
B	Vic	Civil	Disciplinary decision about suitability of ongoing teacher registration (2003)
C	NSW	Civil	Legal challenge of workplace investigation into employee’s conduct (2006)
D^b^	WA	Criminal	Appeal of sentence for conviction of sexual offense/s against child/ren (2007–2008)
E	NSW	Civil	Legal challenge of denied working with children Check (2008–2009)
F	Vic	Criminal	Appeal of sentence for conviction of sexual offense/s against child/ren (2003)
G	WA	Criminal	Appeal of sentence for conviction of sexual offense/s against child/ren (2015–2017)
H	Vic	Civil	Lawsuit of school and former headmaster (2003–2005)
I	Vic	Criminal	Appeal of ruing against application for a permanent stay of the Indictment (1993)
J	Vic	Criminal	Appeal of sentence for conviction of sexual offense/s against child/ren (2007)
K	Qld	Civil	Disciplinary decision about suitability of ongoing teacher registration (2007–2008)
L	Qld	Civil	Disciplinary decision about suitability of ongoing teacher registration (2006)
M	Qld	Civil	Disciplinary decision about suitability of ongoing teacher registration (2014)
N	Vic	Criminal	Appeal of sentence for conviction of sexual offense/s against child/ren (2004)
O	NT	Criminal	Appeal of sentence for conviction of sexual offense/s against child/ren (2010–2011)
P	ACT	Criminal	Sentencing for conviction of sexual offense/s against child/ren (2007)
Q	SA	Criminal	Appeal of sentence for conviction of sexual offense/s against child/ren (2017)
R	Vic	Civil	Disciplinary decision about suitability of ongoing teacher registration (2002)
S^b^	WA	Civil	Legal challenge of denied working with children Check (2007–2008)

^a^Bracketed years denote when perpetration occurred, not the date of legal proceedings.

^b^These two documents depict the same matter but pertain to different systems.

**Table A2.** Characteristics of Sampled Experts.

Pseudonym	Years of experience	Description of experience in educational settings
Claudia (F)	13	Workplace investigations and safeguarding support/advice with schools generally
Daniel (M)	7	Workplace investigations and safeguarding support/advice with schools generally
Kimberly (F)	20	Oversight body, workplace investigations, and safeguarding support/advice with schools generally
Cynthia (F)	21	Personal plaintiff matters, workplace investigations, and safeguarding support/advice with schools generally
Melissa (F)	11	Personal plaintiff matters, workplace investigations, and safeguarding support/advice with schools generally
Kirk (M)	22	Criminal investigations, oversight body, and workplace investigations with schools generally
Tracy (F)	8	Workplace investigations, assessing historical claims, and safeguarding support/advice with schools generally
Dylan (M)	8.5	Workplace investigations in the context of teacher registration and professional standards

*Note.* Further demographic information is omitted to preserve anonymity.
